# Association between hemoglobin A1c and carotid atherosclerosis in rural community-dwelling elderly Japanese men

**DOI:** 10.1186/s40101-015-0054-6

**Published:** 2015-04-01

**Authors:** Yuji Shimizu, Mio Nakazato, Shimpei Sato, Jun Koyamatsu, Hirotomo Yamanashi, Mako Nagayoshi, Koichiro Kadota, Naomi Hayashida, Hironori Yamasaki, Yosuke Kusano, Noboru Takamura, Kiyoshi Aoyagi, Takahiro Maeda

**Affiliations:** Department of Community Medicine, Nagasaki University Graduate School of Biomedical Science, Nagasaki, Japan; Department of Island and Community Medicine, Nagasaki University Graduate School of Biomedical Science, Nagasaki, Japan; Department of Global Health, Medicine and welfare, Atomic bomb Disease Institute, Nagasaki University Graduate School of Biomedical Sciences, Nagasaki, Japan; Center for Health and Community Medicine, Nagasaki University, Nagasaki, Japan; Department of Internal Medicine, Nagasaki National Hospital, Nagasaki, Japan; Department of Public Health, Nagasaki University Graduate School of Biomedical Sciences, Nagasaki, Japan

## Abstract

**Background:**

Recent studies have reported an association between both higher and lower levels of hemoglobin A1c (HbA1c) and higher mortality of diabetes patients. Like diabetes, carotid atherosclerosis is a well known lifestyle-related disease. However, no studies have yet reported an association between HbA1c levels and carotid atherosclerosis.

**Methods:**

We conducted a cross-sectional study of 1,150 Japanese elderly men aged ≥60 years who were undergoing general health checkups. Carotid atherosclerosis was defined as a carotid intima-media thickness (CIMT) ≥1.1 mm. Since body mass index (BMI) is regarded as a cardiovascular risk factor that exerts a strong influence on both HbA1c levels and carotid atherosclerosis, we performed a stratified analysis of this risk based on BMI.

**Results:**

Using the intermediate HbA1c quintile as a reference group, the groups in the lowest HbA1c quintiles showed a significantly higher risk of carotid atherosclerosis in patients with low BMI (≤23 kg/m^2^) *vs*. no increased risk in those with high BMI (>23 kg/m^2^). The association of HbA1c with carotid atherosclerosis became slightly stronger when these analyses were limited to subjects who were not taking glucose-lowering medications or medications for hyperlipidemia and cardiovascular disease. After adjusting for classical cardiovascular risk factors, adjusted odds ratios (ORs) for carotid atherosclerosis were 1.36 (0.84 to 2.20) for total subjects, 2.29 (1.12 to 4.66) for low-BMI groups, and 0.68 (0.33 to 1.41) for high-BMI groups.

**Conclusions:**

Lower HbA1c level is a significant risk factor for carotid atherosclerosis in rural community-dwelling elderly Japanese men with low, but not high BMI, particularly in those not taking glucose-lowering medication.

## Introduction

The exact nature of the association between hemoglobin A1c (HbA1c) and cardiovascular disease remains controversial. Previous studies established the existence of a linear relationship between HbA1c and cardiovascular mortality for type 2 diabetes [1], type 1 diabetes [2], and non-diabetic subjects [3],while other studies found that both low and high mean HbA1c values were associated with increased all-causal mortality and cardiac events in diabetes patients [4,5]. The Norfolk prospective population study detected a J-shaped association between HbA1c concentrations and incidence of stroke [6]. Furthermore, an increase in carotid intima-media thickness (CIMT) was reported to be an independent risk factor for stroke [7]. However, no studies have reported on the association between HbA1c levels and carotid atherosclerosis evaluated by CIMT. Our previous study that investigated the association between atherosclerosis (evaluated using CIMT) and diabetes (defined as HbA1c (NGSP: National Glycohemoglobin Standardization Program) ≥6.5% and/or initiation of glucose-lowering medication or insulin therapy) in a community-based sample of subjects divided into tertiles according to triglycerides-to-HDL cholesterol ratio (TG-HDL) levels reported that only diabetic patients with high TG-HDL but not intermediate and low TG-HDL were at significant risk for atherosclerosis [8]. We also reported a significant inverse association between low-TG-HDL diabetes and body mass index (BMI) and a significant positive association between high-TG-HDL diabetes and BMI in a community-based sample [9]. These studies indicate that BMI status might influence the association between HbA1c and carotid atherosclerosis. We therefore hypothesized that not only higher but also lower HbA1c is a risk factor for carotid atherosclerosis and that BMI status may influence this association. To investigate possible associations, we conducted a cross-sectional study of 1,150 elderly Japanese men aged ≥60 years (range: 60 to 94 years) who were undergoing general health checkups.

## Methods

### Study sample

The original sample included 1,185 men aged 60 to 94 years residing in a rural community on the western Japan Goto Islands. Subjects were recruited between 2005 and 2012. A total of 35 individuals with missing data (6 individuals without BMI data, 3 individuals without blood pressure data, 23 without alcohol consumption data, and 3 individuals without blood test data) were excluded, leaving a total of 1,150 men for enrolment in this study. The mean age of the study sample was 70.1 years (±6.8 SD; range 60 to 94). This study was approved by the Ethics Committee for Human Use of Nagasaki University (project registration number 0501120073).

### Data collection and laboratory measurements

Systolic and diastolic blood pressures at rest in a sitting position were recorded by trained technicians using a blood pressure measuring device (HEM-907; Omron, Kyoto, Japan). Briefly, height in stocking feet and weight in light clothing were measured with an automatic body composition analyzer (BF-220; Tanita, Tokyo, Japan) prior to blood drawing. Trained interviewers obtained information on smoking status (never smoker, former smoker, current smoker), alcohol consumption [non-drinker and current light-to-moderate drinker (one to six times/week), current heavy drinker (every day)], medical history, use of medications for cardiovascular disease, use of medication for hyperlipidemia, use of antihypertensive agents, and use of medications for diabetes mellitus. Fasting blood samples were collected in sodium fluoride tubes and siliconized tubes. Samples from siliconized tubes were used for serum separation and centrifugation following blood coagulation. All measurements were conducted using standard laboratory procedures at SRL, Inc. (Tokyo, Japan). HbA_1C_ was measured with the latex agglutination method [10] using samples from the sodium fluoride tubes. Serum concentration of HDL-cholesterol was measured using the direct method, serum triglycerides (TG) and creatinine were measured using the enzyme method, and aspartate aminotransferase (AST) was measured using the JASCC standardization method. HbA1c (NGSP) level was calculated using the following equation, which was recently proposed by a working group of the Japanese Diabetes Society (JDS): HbA1c(NGSP) = HbA1c(JDS) × 1.02 + 0.25% [11]. The glomerular filtration rate (GFR) was estimated by using an established method with three variations recently proposed by a working group of the Japanese Chronic Kidney Disease Initiative [12], based on which GFR (mL/min/1.73 m^2^) = 194 × (serum creatinine (enzyme method))^−1.094^ × (age)^−0.287^.

### Carotid B-mode ultrasound imaging

Measurement of CIMT by ultrasonography of the left and right carotid arteries was performed by two medical doctors (NT and MN) using a LOGIQ Book XP with a 10-MHz transducer (GE Healthcare, Milwaukee, WI, USA) that was programmed with the IMT measurement software Intimascope (Cross Media Ltd., Tokyo, Japan) [13]. The protocol that was used has been described in detail elsewhere [14]. The values for right and left CIMT without plaque measurement were calculated, and the higher CIMT value was used for the analysis. Based on previous studies [8,15,16], we defined carotid atherosclerosis as a CIMT ≥1.1 mm. Intra-observer variation agreement for CIMT (NT, *n* = 32) was 0.91 (*P* value (*P*) <0.01), and inter-observer variation agreement (NT *vs*. MN, *n* = 41) was 0.78 (*P* < 0.01).

### Statistical analysis

Differences in age-adjusted mean values or prevalence of potential confounding factors in relation to HbA1c levels were calculated using ANOVA or logistic regression models. Odds ratios (ORs) and 95% confidence intervals (CIs) for carotid atherosclerosis (CIMT ≥ 1.1 mm) associated with HbA1c levels were calculated with the aid of logistic regression models. In addition, subjects were stratified by BMI status since a relatively high BMI is regarded as one of the most common cardiovascular risk factors. Since the World Health Organization (WHO) identified BMI ≥23 kg/m^2^, which corresponds to the median BMI values of the men in our study, as an indicator of enhanced risk of disease in Asian populations [17], the BMI cutoff point was set at 23 kg/m^2^. Furthermore, analyses were restricted to subjects who were not taking glucose-lowering medications or medications for hyperlipidemia and cardiovascular disease. Two different approaches were used to adjust for confounding factors. The first was adjustment only for age. For the second approach, we included other potential confounding factors such as smoking status, alcohol consumption, systolic blood pressure (mmHg), BMI (kg/m^2^), antihypertensive medication use (no, yes), taking glucose-lowering medication (no, yes), taking medication for hyperlipidemia (no, yes), taking medication for cardiovascular disease (no, yes), HDL-cholesterol (mg/dL), TG (mg/dL), AST (IU/L), and GFR (mL/min/1.73 m^2^). Because glucose-lowering medication and taking medication for hyperlipidemia and cardiovascular disease might strongly confound these associations [7,18,19], we restricted further analysis to subjects not taking glucose-lowering medication or medication for hyperlipidemia and cardiovascular disease. All statistical analyses were performed with the SAS system for Windows (version 9.3; SAS Inc., Cary, NC). All *P* values (*P*) and *P* for the trend (*P*) for statistical tests were two-tailed, with values of <0.05 regarded as being statistically significant.

## Results

### Clinical characteristics of the subjects

The clinical characteristics of the subjects in this study are summarized in Table 1. HbA1c levels were significantly and inversely associated with serum creatinine and significantly and positively associated with GFR for total subjects and those with low or high BMI. Additionally, a J-shaped association between HbA1c levels and history of cardiovascular disease was observed for total subjects and those with low, but not high BMI.

**Table 1:** **Characteristics of the study population based on hemoglobin A**_**1C**_ **(HbA1c) level quintiles**

	**HbA**_**1c**_ **level quintiles**					
	**Q1 (low)**	**Q2**	**Q3**	**Q4**	**Q5 (high)**	***P***
Hemoglobin A_1c_ (HbA_1c_) median values, %	5.04	5.35	5.45	5.76	6.17	
Total subjects						
No. at risk	190	205	258	246	251	
Age, year	70.3 ± 6.3	70.5 ± 7.6	70.3 ± 6.7	70.7 ± 7.0	70.3 ± 7.0	
Systolic blood pressure, mmHg	144 ± 20	147 ± 22	143 ± 19	143 ± 19	146 ± 22	0.107
Antihypertensive medication use, %	35.8	30.2	38.8	35.8	40.6	0.165
Body mass index, kg/m^2^	22.8 ± 2.8	23.5 ± 3.1	23.1 ± 2.8	23.9 ± 2.9	24.2 ± 3.4	<0.001
Current drinker, %	53.2	52.2	51.6	42.3	44.2	0.058
Current smoker, %	20.5	17.6	24.0	17.5	19.5	0.381
Serum triglycerides, mg/dL	98 ± 50	119 ± 80	105 ± 58	129 ± 82	134 ± 98	<0.001
Serum HDL-cholesterol, mg/dL	56 ± 15	54 ± 14	56 ± 15	53 ± 14	53 ± 15	0.059
Serum aspartate aminotransferase (AST), IU/L	25 ± 12	25 ± 9	24 ± 7	25 ± 8	27 ± 13	0.033
Glucose-lowering medication use, %	2.1	2.0	1.2	2.8	27.9	<0.001
Lipid-lowering medication use, %	3.2	5.9	5.4	7.7	10.8	0.020
History of cardiovascular disease, %	9.5	8.3	12.4	15.9	16.3	0.033
Taking medication for cardiovascular disease, %	7.4	7.3	11.2	14.6	14.3	0.025
Serum creatinine, mg/dL	0.98 ± 0.32	0.92 ± 0.28	0.92 ± 0.29	0.88 ± 0.22	0.88 ± 0.22	<0.001
Glomerular filtration rate (GFR), mL/min/1.73 m^2^	63.7 ± 17.9	67.2 ± 17.6	67.6 ± 17.2	69.5 ± 16.4	70.5 ± 16.8	<0.001
Low body mass index (BMI)						
No. at risk	103	102	127	98	90	
Age, year	71.5 ± 6.7	71.5 ± 8.1	70.2 ± 7.2	71.6 ± 7.3	70.7 ± 7.2	
Systolic blood pressure, mmHg	140 ± 21	142 ± 20	140 ± 18	138 ± 17	140 ± 22	0.543
Antihypertensive medication use, %	28.2	25.5	29.1	23.5	25.6	0.778
Body mass index, kg/m^2^	20.8 ± 1.5	20.9 ± 1.5	20.8 ± 1.7	21.1 ± 1.5	20.7 ± 1.6	0.429
Current drinker, %	60.2	50.0	50.2	48.2	49.0	0.004
Current smoker, %	23.3	20.6	29.9	21.4	21.1	0.524
Serum triglycerides, mg/dL	90 ± 52	101 ± 55	93 ± 50	107 ± 66	103 ± 66	0.175
Serum HDL-cholesterol, mg/dL	60 ± 15	56 ± 14	58 ± 15	56 ± 15	57 ± 16	0.370
Serum aspartate aminotransferase (AST), IU/L	26 ± 14	25 ± 9	23 ± 5	25 ± 9	25 ± 10	0.328
Glucose-lowering medication use, %	3.9	2.9	0.8	10.2	30.0	<0.001
Lipid-lowering medication use, %	2.9	5.9	4.7	8.2	5.6	0.573
History of cardiovascular disease, %	7.8	4.9	8.7	14.3	16.7	0.032
Taking medication for cardiovascular disease, %	5.8	3.9	7.8	13.3	12.2	0.068
Serum creatinine, mg/dL	1.00 ± 0.37	0.94 ± 0.35	0.91 ± 0.34	0.85 ± 0.16	0.88 ± 0.25	0.006
GFR, mL/min/1.73 m^2^	62.4 ± 17.0	68.0 ± 20.6	69.6 ± 18.5	71.1 ± 14.7	70.1 ± 17.3	0.004
High BMI						
No. at risk	87	103	131	148	161	
Age, year	68.9 ± 5.6	69.5 ± 6.9	70.4 ± 6.1	70.2 ± 6.8	70.1 ± 7.2	
Systolic blood pressure, mmHg	149 ± 17	152 ± 23	147 ± 19	146 ± 20	150 ± 21	0.154
Antihypertensive medication use, %	44.8	35.0	48.1	43.9	49.1	0.258
Body mass index, kg/m^2^	25.1 ± 1.9	26.0 ± 2.1	25.4 ± 1.7	25.7 ± 1.8	26.1 ± 2.5	0.001
Current drinker, %	44.8	54.4	55.0	46.6	47.2	0.296
Current smoker, %	17.2	14.6	18.3	14.9	18.6	0.822
Serum triglycerides, mg/dL	106 ± 45	137 ± 96	116 ± 62	143 ± 88	152 ± 108	<0.001
Serum HDL-cholesterol, mg/dL	51 ± 14	52 ± 15	54 ± 14	51 ± 12	51 ± 13	0.300
Serum aspartate aminotransferase (AST), IU/L	24 ± 10	26 ± 10	24 ± 8	25 ± 8	28 ± 15	0.021
Glucose-lowering medication use, %	0.0	1.0	1.5	4.1	26.7	<0.001
Lipid-lowering medication use, %	3.4	5.8	6.1	7.4	13.7	0.028
History of cardiovascular disease, %	11.5	11.7	16.0	16.9	16.1	0.770
Taking medication for cardiovascular disease, %	9.2	10.7	14.5	15.5	15.5	0.645
Serum creatinine, mg/dL	0.96 ± 0.26	0.91 ± 0.18	0.93 ± 0.23	0.91 ± 0.25	0.87 ± 0.20	0.021
GFR, mL/min/1.73 m^2^	65.3 ± 18.8	66.5 ± 14.0	65.6 ± 15.5	68.4 ± 17.4	70.5 ± 16.2	0.021

Low BMI consists of BMI <23 kg/m^2^ and high BMI consists of BMI ≥23 kg/m^2^. *P*: age-adjusted values of *P* for the trend.

### Association between carotid atherosclerosis and HbA1c

Table 2 shows ORs and 95% CIs for carotid atherosclerosis in relation to HbA1c levels for the study sample. Using the data for the median HbA1c level quintile as reference, lower HbA1c level was found to be a significant risk factor for carotid atherosclerosis in subjects with low BMI but not with high BMI, and no significant association was noted even when HbA1c levels were at their highest. With the median HbA1c level (Q3) as the reference group, the multi-adjusted ORs (95% CI) for carotid atherosclerosis in subjects with low BMI were 1.78 (0.92 to 3.44) for Q1 and 2.05 (1.06 to 3.96) for Q2, while for subjects with high BMI, the corresponding values were 0.66 (0.35 to 1.27) and 0.94 (0.51 to 1.73), respectively.

**Table 2:** **Carotid atherosclerosis in relation to hemoglobin A1c (HbA1c) levels**

	**HbA1c level quintiles**				
	**Q1 (low)**	**Q2**	**Q3**	**Q4**	**Q5 (high)**
Total subjects					
No. at risk	190	205	258	246	251
No. of cases (percentage)	57 (30.0)	64 (31.2)	67 (26.0)	71 (28.9)	83 (33.1)
Age-adjusted odds ratio (OR)	1.24 (0.81 to 1.90)	1.28 (0.84 to 1.95)	1.00	1.13 (0.76 to 1.68)	1.42 (0.94 to 2.10)
Multivariable OR	1.21 (0.78 to 1.88)	1.36 (0.88 to 2.11)	1.00	1.13 (0.82 to 2.01)	1.13 (0.74 to 1.71)
Low BMI					
No. at risk	103	102	127	98	90
No. of cases (percentage)	35 (34.0)	35 (34.3)	25 (19.7)	27 (27.6)	21 (23.3)
Age-adjusted OR	2.01 (1.08 to 3.73)	2.01 (1.07 to 3.75)	1.00	1.44 (0.76 to 2.72)	1.23 (0.63 to 2.40)
Multivariable OR	1.78 (0.92 to 3.44)	2.05 (1.06 to 3.96)	1.00	1.55 (0.77 to 3.09)	1.26 (0.58 to 2.76)
High BMI					
No. at risk	87	103	131	148	161
No. of cases (percentage)	22 (25.3)	29 (28.2)	42 (32.1)	44 (29.7)	62 (38.5)
Age-adjusted OR	0.78 (0.42 to 1.45)	0.86 (0.49 to 1.53)	1.00	0.90 (0.54 to 1.51)	1.35 (0.83 to 2.22)
Multivariable OR	0.66 (0.35 to 1.27)	0.94 (0.51 to 1.73)	1.00	0.91 (0.53 to 1.55)	1.26 (0.72 to 2.22)

Multivariable: adjusted further for age, systolic blood pressure, antihypertensive medication use, body mass index, smoking, alcohol intake, taking medication for cardiovascular disease, use of glucose-lowering medication, lipid-lowering medication use, serum triglyceride (TG), serum HDL-cholesterol, serum aspartate aminotransferase (AST), and estimated glomerular filtration rate (GFR). Low body mass index (BMI) consists of BMI <23 kg/m^2^ and high BMI consists of BMI ≥23 kg/m^2^.

### Clinical characteristics limited to subjects with HbA1c (≤Q3)

We also investigated the effects of interaction between HbA1c and two BMI categories, low BMI and high BMI, on carotid atherosclerosis. Significant interaction was seen between HbA1c and BMI status among subjects with HbA1c (≤Q3) but not with HbA1c (≥Q3), with the multivariable-adjusted *P* values for the effect of this interaction on carotid atherosclerosis of *P* = 0.027 for subjects with HbA1c (≤Q3) and *P* = 0.951 for HbA1c (≥Q3).

Since the significant interaction between HbA1c and the two BMI categories on carotid atherosclerosis was observed only among subjects with HbA1c (≤Q3) but not with HbA1c (≥Q3), we conducted a further analysis to evaluate the age-adjusted clinical characteristics limited to subjects with HbA1c (≤Q3) (Table 3). Significant positive association between HbA1c levels and GFR values was limited to subjects with low BMI. For these subjects, the age-adjusted mean value of GFR for each HbA1c level was 62.7 mL/min/1.73 m^2^ for Q1, 68.4 mL/min/1.73 m^2^ for Q2, and 69.0 mL/min/1.73 m^2^ for Q3 (*P* = 0.020), while for subjects with high BMI, the corresponding values were 64.8 mL/min/1.73 m^2^, 66.4 mL/min/1.73 m^2^, and 66.0 mL/min/1.73 m^2^ (*P* = 0.792), respectively.

**Table 3:** **Age-adjusted characteristics of the study population limited to subjects with HbA1c (≤Q3)**

	**Total subjects**				**Low BMI**				**High BMI**			
	**HbA**_**1c**_ **level quintiles**				**HbA**_**1c**_ **level quintiles**				**HbA**_**1c**_ **level quintiles**			
	**Q1 (low)**	**Q2**	**Q3 (high)**	***P***	**Q1 (low)**	**Q2**	**Q3 (high)**	***P***	**Q1 (low)**	**Q2**	**Q3 (high)**	***P***
No. at risk	190	205	258		103	102	127		87	103	131	
Age, year	70.3 ± 6.3	70.5 ± 7.6	70.3 ± 6.7		71.5 ± 6.7	71.5 ± 8.1	70.2 ± 7.2		68.9 ± 5.6	69.5 ± 6.9	70.4 ± 6.1	
Systolic blood pressure, mmHg	144	147	143	0.187	140	142	140	0.721	149	152	147	0.223
Antihypertensive medication use, %	35.9	30.1	38.9	0.132	27.4	24.8	30.3	0.642	46.1	35.2	47.0	0.144
Body mass index, kg/m^2^	22.8	23.5	23.1	0.062	20.8	20.9	20.8	0.674	25.1	26.0	25.4	0.008
Current drinker, %	53.1	52.3	51.5	0.942	60.6	50.4	47.4	0.121	43.7	54.1	55.9	0.179
Current smoker, %	20.5	17.6	24.0	0.242	23.8	21.0	29.2	0.334	16.8	14.5	18.7	0.692
Serum triglycerides, mg/dL	97	119	105	0.003	91	101	92	0.284	105	137	117	0.010
Serum HDL-cholesterol, mg/dL	56	54	56	0.319	60	56	58	0.157	51	52	54	0.321
Serum aspartate aminotransferase (AST), IU/L	25	25	24	0.263	26	25	23	0.127	24	26	24	0.322
Glucose-lowering medication use, %	2.1	2.0	1.2	0.699	3.9	3.0	0.8	0.279	0.0	1.0	1.6	0.481
Lipid-lowering medication use, %	3.2	5.9	5.4	0.410	2.9	5.9	4.7	0.589	3.4	5.8	6.1	0.659
History of cardiovascular disease, %	9.5	8.2	12.4	0.302	7.6	4.7	8.9	0.471	12.2	11.8	15.4	0.675
Taking medication for cardiovascular disease, %	7.4	7.3	11.3	0.218	5.6	3.7	8.2	0.354	9.8	10.8	14.0	0.585
Serum creatinine, mg/dL	0.98	0.92	0.92	0.055	1.00	0.93	0.92	0.193	0.96	0.91	0.93	0.334
Glomerular filtration rate (GFR), mL/min/1.73 m^2^	63.7	67.3	67.5	0.036	62.7	68.4	69.0	0.020	64.8	66.4	66.0	0.792

Age: mean ± standard deviation. Low BMI consists of BMI <23 kg/m^2^ and high BMI consists of BMI ≥23 kg/m^2^. *P*: age-adjusted values of *P* for the trend. BMI: body mass index.

Although no significant associations were observed in the analysis with regard to HbA1c levels and current-drinker status, an inverse association was observed for subjects with low BMI, whereas a positive association was observed for subjects with high BMI. The age-adjusted prevalence of current-drinker status for each HbA1c level was 60.6% for Q1, 50.4% for Q2, and 47.4% for Q3 (*P* = 0.121), while for subjects with high BMI, the corresponding values were 43.7%, 54.1%, and 55.9% (*P* = 0.179), respectively.

### Association between carotid atherosclerosis and HbA1c in subjects not taking glucose-lowering medication or medication for hyperlipidemia and cardiovascular disease

We also investigated the associations in subjects who had not taken glucose-lowering medication or medication for hyperlipidemia and cardiovascular disease (Table 4). For subjects with HbA1c (≤Q3), the association between HbA1c level and carotid atherosclerosis became slightly stronger. With median HbA1c level (Q3) as the reference group, the multi-adjusted ORs (95%CI) of carotid atherosclerosis for subjects with low BMI were 2.29 (1.12 to 4.66) for Q1 and 2.15 (1.05 to 4.39) for Q2, while for subjects with high BMI, the corresponding values were 0.68 (0.33 to 1.41) and 1.09 (0.55 to 2.16), respectively.

**Table 4:** **Carotid atherosclerosis in relation to hemoglobin A1c (HbA1c) levels in subjects not taking medication**

	**HbA**_**1c**_ **level quintiles**				
	**Q1 (low)**	**Q2**	**Q3**	**Q4**	**Q5 (high)**
Total subject					
No. at risk	168	180	216	192	138
No. of cases (percentage)	51 (30.4)	54 (30.0)	51 (23.6)	54 (28.1)	46 (33.3)
Age-adjusted odds ratio (OR)	1.42 (0.89 to 2.26)	1.35 (0.85 to 2.13)	1.00	1.26 (0.80 to 1.98)	1.62 (1.00 to 2.63)
Multivariable OR	1.36 (0.84 to 2.20)	1.44 (0.89 to 2.33)	1.00	1.30 (0.80 to 2.10)	1.58 (0.94 to 2.66)
Low body mass index (BMI)					
No. at risk	92	91	111	78	50
No. of cases (percentage)	33 (35.9)	29 (31.9)	20 (18.0)	19 (24.4)	10 (20.0)
Age-adjusted OR	2.40 (1.24 to 4.67)	2.04 (1.03 to 4.01)	1.00	1.42 (0.69 to 2.91)	1.13 (0.48 to 2.67)
Multivariable OR	2.29 (1.12 to 4.66)	2.15 (1.05 to 4.39)	1.00	1.50 (0.67 to 3.33)	0.99 (0.39 to 2.53)
High BMI					
No. at risk	76	89	105	114	88
No. of cases (percentage)	18 (23.7)	25 (28.1)	31 (29.5)	35 (30.7)	36 (40.9)
Age-adjusted OR	0.80 (0.41 to 1.59)	0.93 (0.49 to 1.74)	1.00	1.07 (0.59 to 1.92)	1.66 (0.90 to 3.05)
Multivariable OR	0.68 (0.33 to 1.41)	1.09 (0.55 to 2.16)	1.00	1.19 (0.64 to 2.21)	2.01 (1.04 to 3.90)

Multivariable: adjusted further for age, systolic blood pressure, antihypertensive medication use, body mass index, smoking, alcohol intake, serum triglyceride (TG), serum HDL-cholesterol, serum aspartate aminotransferase (AST), and estimated glomerular filtration rate (GFR). Low BMI consists of BMI <23 kg/m^2^ and high BMI consists of BMI ≥23 kg/m^2^.

The multivariable-adjusted *P* values for the corresponding interaction with carotid atherosclerosis were *P* = 0.020 for subjects with HbA1c (≤Q3) and *P* = 0.775 for those with HbA1c (≥Q3).

## Discussion

Our findings demonstrate that lower HbA1c levels constitute a significant risk for carotid atherosclerosis in subjects with low BMI but not in those with high BMI. These associations were also observed in subjects who did not take glucose-lowering medication.

Our previous study reported a significant positive association between BMI and patients with diabetes of the type that carries a risk of atherosclerosis and an inverse association in patients with diabetes of the type that does not pose such a risk [9]. This is compatible with the finding from our present study that among subjects not taking glucose-lowering medication or medication for hyperlipidemia and cardiovascular disease, the highest HbA1c levels (Q5) showed a significant risk of carotid atherosclerosis in subjects with high BMI but not in those with low BMI. However, no significant interaction between HbA1c and BMI status with regard to carotid atherosclerosis was observed in the HbA1c (≥Q3) group as a result of this higher risk of carotid atherosclerosis with higher HbA1c levels (Q4) in subjects with low BMI but not in subjects with high BMI. To clarify the influence of BMI on the association between carotid atherosclerosis and HbA1c among subjects with HbA1c (≥Q3), further investigation with a larger population is necessary.

Mechanisms explaining why lower HbA1c levels lead to a significant risk of atherosclerosis have not been elucidated. A previous study on type 1 diabetes reported episodes of hypoglycemia (<70 mg/dL) as a potential aggravating factor for preclinical atherosclerosis [20], and repeated episodes of hypoglycemia may perform a crucial role for this association. Since another study reported that chronic kidney disease is a risk factor for hypoglycemia (<70 mg/dL) and this association was observed both in subjects with or without diabetes [21], those with reduced GFR may demonstrate a frequency of hypoglycemia that results in lower HbA1c levels. Further investigations we had conducted on subjects with HbA1c (≤Q3) showed a significant inverse association between estimated GFR and HbA1c in subjects with low BMI but not in those with high BMI. On the other hand, moderate low-glucose exposure rapidly impairs endothelial function [22], and endothelial dysfunction has been recognized as one of the initial mechanisms leading to glomerular injury [23] and atherosclerosis. Lower HbA1c levels may be associated both with carotid atherosclerosis and reduced GFR by indicating a higher frequency of low-glucose exposure. A previous study reporting that not only higher but also lower levels of HbA1c are associated with a higher risk of death for subjects with diabetes and chronic kidney disease partially supports our results [5]. However, the reason why this significant association of lower HbA1c with carotid atherosclerosis was restricted to subjects with low BMI has not yet been clarified. Subjects with low-BMI and low-HbA1c level might have a higher risk of hypoglycemia compared to subjects with high BMI and low HbA1c. Further investigations are necessary to clarify these associations.

Since the association between regular alcohol intake and incidence of carotid atherosclerosis was reported to be J-shaped (with light drinkers facing a lower risk than either heavy drinkers or abstainers) [24], alcohol intake might also influence the association between HbA1c level and carotid atherosclerosis. In our study, even though the statistical power did not reach the level of significance, HbA1c levels were inversely associated with current-drinker status in subjects with low BMI but not in subjects with high BMI. Further investigations using detailed alcohol consumption data are necessary.

### Limitations

Potential limitations in our study warrant consideration. Even though we found in a study limited to subjects not taking glucose-lowing medication or medication for hyperlipidemia and cardiovascular disease that the highest HbA1c levels represent a significant risk for carotid atherosclerosis in subjects with high but not with low BMI, the interaction between height and BMI category for these subjects did not reach the level of significance, with a multivariable adjusted *P* value of 0.474 for the effect of this interaction on carotid atherosclerosis. Further studies using larger numbers of subjects will be necessary to determine the reason for this finding. Moreover, we could not evaluate the frequency of hypoglycemia for the low-HbA1c group, which might have been higher in subjects with low BMI than in those with high BMI. A further clinical study using daily blood sugar data to evaluate daily variations will thus be necessary. Since we did not have detailed data on medications for cardiovascular diseases such as antithrombotic therapy and/or anticoagulation therapy, which might have a strong effect on the progression of atherosclerosis, adjustments for these medications are of limited value. However, further investigations restricted to subjects who were not taking medication for cardiovascular disease showed significant associations. Finally, because this was a cross-sectional study, no causal relationships could be established.

## Conclusion

In conclusion, lower HbA1c levels constitute a significant risk for carotid atherosclerosis in subjects with low BMI but not in subjects with high BMI as shown in rural community-dwelling elderly Japanese men, particularly in those that do not take glucose-lowering medications.

## Consent

Written consent forms were available in Japanese to ensure comprehensive understanding of the study objectives, and informed consent was signed or thumb printed by the participants.

## Abbreviations

AST: aspartate aminotransferase
BMI: body mass index
CI: confidence intervals
CIMT: carotid intima-media thickness
GFR: glomerular filtration rate
HbA1c: hemoglobin A1C
JDS: Japanese Diabetes Society
NGSP: National Glycohemoglobin Standardization Program
ORs: odds ratios
TG: triglycerides

## References

[1] Eeg-Olofsson K, Cederholm J, Nilsson PM, Zethelius B, Svensson AM, Gudbjörnsdóttir S (2010). New aspect of HbA1c as a risk factor for cardiovascular disease in type 2 diabetes: an observational study from the Swedish National Diabetes Register (NDR). J Intern Med.

[2] Eeg-Olofsson K, Cederholm J, Nilsson PM, Zethelius B, Svensson AM, Gudbjörnsdóttir S (2010). Glycemic control and cardiovascular disease in 7,454 patients with type 1 diabetes: an observational study from the Swedish National Diabetes Register (NDR). Diabetes Care.

[3] Barr EL, Boyko EJ, Zimmet PZ, Wolfe R, Tonkin AM, Shaw JE (2009). Continuous relationships between non-diabetic hyperglycemia and both cardiovascular disease and all-cause mortality: the Australian Diabetes, Obesity, and lifestyle (AusDiab) study. Diabetelogia.

[4] Currie CJ, Peters JR, Tynan A, Evans M, Heine RJ, Bracco OL (2010). Survival as a function of HbA(1c) in people with type 2 diabetes: a retrospective cohort study. Lancet.

[5] Shurraw S, Hemmelgarn B, Lin M, Majumdar SR, Klarenbach S, Manns B (2011). Alberta Kidney Disease Network: association between glycemic control and adverse outcomes in people with diabetes mellitus and chronic kidney disease: a population-based cohort study. Arch Intern Med.

[6] Myint PK, Shinha S, Wareham NJ, Bingham SA, Luben RN, Welch AA (2007). Glycated hemoglobin and risk of stroke in people without known diabetes in the European Prospective Investigation into Cancer (EPIC)-Norfolk prospective population study: a threshold relationship?. Stroke.

[7] Kitamura A, Iso H, Imano H, Ohira T, Okada T, Sato S (2004). Carotid intima-media thickness and plaque characteristics as a risk factor for stroke in Japanese elderly men. Stroke.

[8] Shimizu Y, Nakazato M, Sekita T, Kadota K, Yamasaki H, Takamura N (2013). Association of arterial stiffness and diabetes with triglycerides-to-HDL cholesterol ratio for Japanese men: The Nagasaki Islands Study. Atherosclerosis.

[9] Shimizu Y, Nakazato M, Sekita T, Kadota K, Sato S, Koyamatsu J (2013). Body mass index and triglyceride-to-HDL-cholesterol ratio in relation to risk of diabetes: The Nagasaki Islands study. Acta Med Nagasaki.

[10] Schnedl WJ, Reisinger EC, Pieber TR, Lipp RW, Schreiber F, Hopmeier P (1995). Hemoglobin Sherwood Forest detected by high performance liquid chromatography for hemoglobin A1c. Am J Clin Pathol.

[11] Kashiwagi A, Kasuga M, Araki E, Oka Y, Hanafusa T, Ito H (2012). Committee on the Standardization of Diabetes Mellitus-Related Laboratory Testing of Japan Diabetes Society. International clinical harmonization of glycated hemoglobin in Japan: From Japanese Diabetes Society to National Glycohemoglobin Standardization Program values. J Diabetes Investig.

[12] Imai E (2008). Equation for estimating GFR from creatinine in Japan. Nihon Rinsho.

[13] Yanase T, Nasu S, Mukuta Y, Shimizu Y, Nishihara T, Okabe T (2006). Evaluation of a new carotid intima-media thickness measurement by B-mode ultrasonography using an innovative measurement software. Intimascope Am J Hypertens.

[14] Hara T, Takamura N, Akashi S, Nakazato M, Maeda T, Wada M (2006). Evaluation of clinical markers of atherosclerosis in young and elderly Japanese adults. Clin Chem Lab Med.

[15] Kawamori R, Yamasaki Y, Matsushima H, Nishizawa H, Nao K, Hougaku H (1992). Prevalence of carotid atherosclerosis in diabetic patients. Ultrasound high-resolution B-mode imaging on carotid arteries. Diabetes Care.

[16] Shimizu Y, Nakazato M, Sekita T, Kadota K, Arima K, Yamasaki H (2013). Relationship between adult height and body weight and risk of carotid atherosclerosis assessed in terms of carotid intima-media thickness: the Nagasaki Islands study. J Physiol Anthropol.

[17] WHO Expert Consultation (2004). Appropriate body-mass index for Asian population and its implications for policy and intervention strategies. Lancet.

[18] Huang ES, Meigs JB, Singer DE (2001). The effect of interventions to prevent cardiovascular disease in patients with type 2 diabetes mellitus. Am J Med.

[19] Mannami T, Konishi M, Baba S, Nishi N, Terao A (1997). Prevalence of asymptomatic carotid atherosclerotic lesions detected by high-resolution ultrasonography and its relation to cardiovascular risk factors in the general population of a Japanese city: the Suita study. Stroke.

[20] Giménez M, Gilabert R, Monteagudo J, Alonso A, Casamitjana R, Paré C (2011). Repeated episodes of hypoglycemia as a potential aggravating factor for preclinical atherosclerosis in subjects with type 1 diabetes. Diabetes Care.

[21] Moen MF, Zhan M, Hsu VD, Walker LD, Einhorn LM, Seliger SL (2009). Frequency of hypoglycemia and its significance in chronic kidney disease. Clin J Am Soc Nephrol.

[22] Wang J, Alexanian A, Ying R, Kizhakekuttu TJ, Dharmashankar K, Vasquez-Vivar J (2012). Acute exposure to low glucose rapidly induces endothelial dysfunction and mitochondrial oxidative stress: role for AMP kinase. Arterioscler Thromb Vasc Biol.

[23] Endemann DH, Schiffrin EL (2004). Endothelial dysfunction. J Am Soc Nephrol.

[24] Kiechl S, Willeit J, Rungger G, Egger G, Oberhollenzer F, Bonora E (1998). Alcohol consumption and atherosclerosis: what is the relation? Prospective results from the Bruneck Study. Stroke.

